# Phosphorylation sites of microtubule-associated protein 1B (MAP 1B) are involved in axon growth and regeneration

**DOI:** 10.1186/s13041-019-0510-z

**Published:** 2019-11-11

**Authors:** Yuya Ishikawa, Masayasu Okada, Atsuko Honda, Yasuyuki Ito, Atsushi Tamada, Naoto Endo, Michihiro Igarashi

**Affiliations:** 1 Division of Orthopedic Surgery, Department of Regenerative and Transplant Medicine, Graduate School of Medical and Dental Sciences, Niigata, Japan; 2 Department of Neurochemistry and Molecular Cell Biology, Graduate School of Medical and Dental Sciences, Niigata University, 1-757 Asahimachi, Chuo-ku, Niigata, 951-8510 Japan; 3 Trans-disciplinary Research Programs, Brain Research Institute, Niigata University, Niigata, Japan; 4 Department of Neurosurgery, Brain Research Institute, Niigata University, Niigata, Japan; 5 Department of iPS Cell Applied Medicine, Kansai Medical University, Hirakata, Osaka 573-1010 Japan

**Keywords:** Phosphorylation, MAP 1B, Development, Growth cone, Axon regeneration

## Abstract

The growth cone is a specialized structure that forms at the tip of extending axons in developing and regenerating neurons. This structure is essential for accurate synaptogenesis at developmental stages, and is also involved in plasticity-dependent synaptogenesis and axon regeneration in the mature brain. Thus, understanding the molecular mechanisms utilized by growth cones is indispensable to understanding neuronal network formation and rearrangement. Phosphorylation is the most important and commonly utilized protein modification in signal transduction. We previously identified microtubule-associated protein 1B (MAP 1B) as the most frequently phosphorylated protein among ~ 1200 phosphorylated proteins. MAP 1B has more than 10 phosphorylation sites that were present more than 50 times among these 1200 proteins. Here, we produced phospho-specific antibodies against phosphorylated serines at positions 25 and 1201 of MAP 1B that specifically recognize growing axons both in cultured neurons and in vivo in various regions of the embryonic brain. Following sciatic nerve injury, immunoreactivity with each antibody increased compared to the sham operated group. Experiments with transected and sutured nerves revealed that regenerating axons were specifically recognized by these antibodies. These results suggest that these MAP 1B phosphorylation sites are specifically involved in axon growth and that phospho-specific antibodies against MAP 1B are useful markers of growing/regenerating axons.

## Introduction

The growth cone is a specialized motile structure that forms at the tip of growing axons of developing neurons and plays a role in accurate synaptogenesis for neuronal network construction [1]. The molecular basis of the mammalian growth cone is poorly understood due to its high complexity. However, recent approaches using proteomics, which quantitatively identifies proteins [2, 3], have gradually contributed to new views of axon growth (for example, [4–6]).

Microtubule-associated protein 1B (MAP 1B) [7–9] functions as a microtubule (MT)-stabilizing protein in developing neurons [10–12] and is highly expressed at various stages of axogenesis [13, 14]. MAP 1B interacts with actin and other regulators of MTs [15–17]. Among microtubule-associated proteins, MAP 1B is the most abundant cytoskeletal protein in the growth cone, as identified by proteomics, except for tubulin and actin [1–3]. In addition, phosphorylation of MAP 1B is involved in axon growth/regeneration and plasticity [18, 19]. Thus, identification of MAP 1B phosphorylation sites and investigation of their roles in axon formation should contribute to the understanding of nerve growth/regeneration mechanisms.

Phosphoproteomics is a new method for comprehensive identification of the phosphorylation sites of proteins [20]. We recently reported results of a phosphoproteomics study of the growth cone membrane (GCM) and revealed that the most frequent phosphorylation sites in GCM are in MAP 1B [21]. Two proline-directed sites for phosphorylation, S25 and S1201, in MAP 1B are the most abundant in MAP 1B, and are also highly frequent among the total phosphorylated sites of ~ 1200 proteins.

Here, we focused on these two sites and produced phospho-specific antibodies (Abs) against them. Both sites were regulated during development, and the Abs recognized growing axons in vivo in various regions of the developing mouse brain. In addition, immunoreactivity for S25 and S1201 also emerged as early as 6 h after sciatic nerve injury and in distally regenerating axons that have extended past the injury point.

Taken together, we conclude that these sites are closely related to axon growth and regeneration, and that the Abs are potential molecular markers of growing/regenerating axons.

## Results

### Both pS25 and pS1201 abs recognized growing axons in the developing brain

We produced phospho-specific Abs against MAP 1B phospho-peptides (Additional file 1: Figure S1A). Mutated peptides including S25A or S1201A were not recognized by the phospho-S25 (pS25) or the phospho-S1201 (pS1201) Abs, respectively (Additional file 1: Figure S1B), indicating that these Abs specifically reacted with phosphorylated S25 and S1201, respectively.

**Fig. 1:**
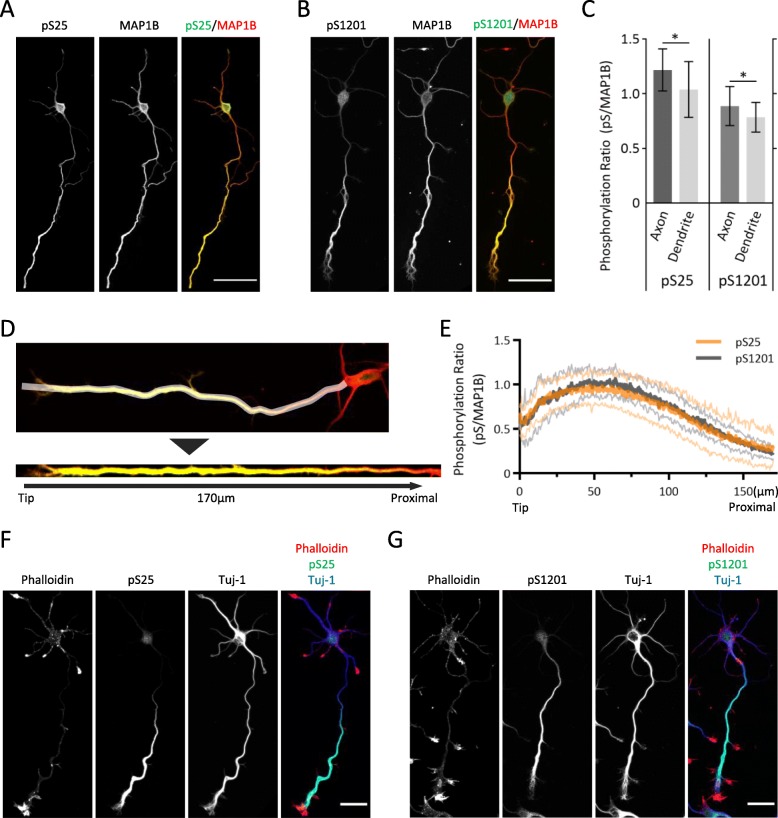
pS25 and pS1201 Abs labeled the growing axons in the cultured neurons. **a** and **b** Immunofluorescent studies of the cultured mouse cortical neuron using pS25 or pS1201 Abs (*green*) with MAP 1B Ab (*red*). Each phospho-specific Ab showed the preferentially stronger immunoreactivity to the axon, than MAP 1B Ab itself. Scale bar: 50 μm. **c** The ratio of the mean signal intensity (pS25 or pS1201 vs MAP 1B) of the axon and the dendrites in each neuron were compared. Data are shown as mean ± SD; *n* = 10; *p < 0.05 by paired *t* test. **d** The white area indicates the region of interest. From the axonal tip to 170 μm proximal, the signal intensity was measured. **e** Profiles of the ratio of the fluorescence intensity (pS25/MAP 1B and pS1201/MAP 1B) from the axonal tip to the proximal part of the axon were comparable. Thick lines indicate mean values, and thin lines indicate SD; *N* = 10. **f** and **g** Immunostaining of cultured mouse cortical neurons using pS25 or pS1201 Abs (*green*) with rhodamine phalloidin for F-actin (*red*) and Tuj-1 Ab for β-III tubulin (*blue*). pS25 and pS1201 Abs labeled the tubulin-positive area of the axon more intensely than the actin-positive area. Scale bar: 25 μm

pS25 (Fig. 1a) and pS1201 (Fig. 1b) Abs preferentially labeled the axons of cultured neurons, and each Ab showed stronger immunoreactivity to the axon than the MAP 1B Ab (Fig. 1c). We measured the intensity of the distal portion of the axon after linearizing the axon (Fig. 1d), and the ratios to MAP 1B itself were calculated. The intensities of pS25 and pS1201 immunoreactivity distally along the axon were similar to each other (Fig. 1e). pS25 or pS1201 immunoreactivity was colocalized with MTs, rather than F-actin, and these Abs recognized the distal axon of the growing neurons (Fig. 1f-g).

**Fig. 2:**
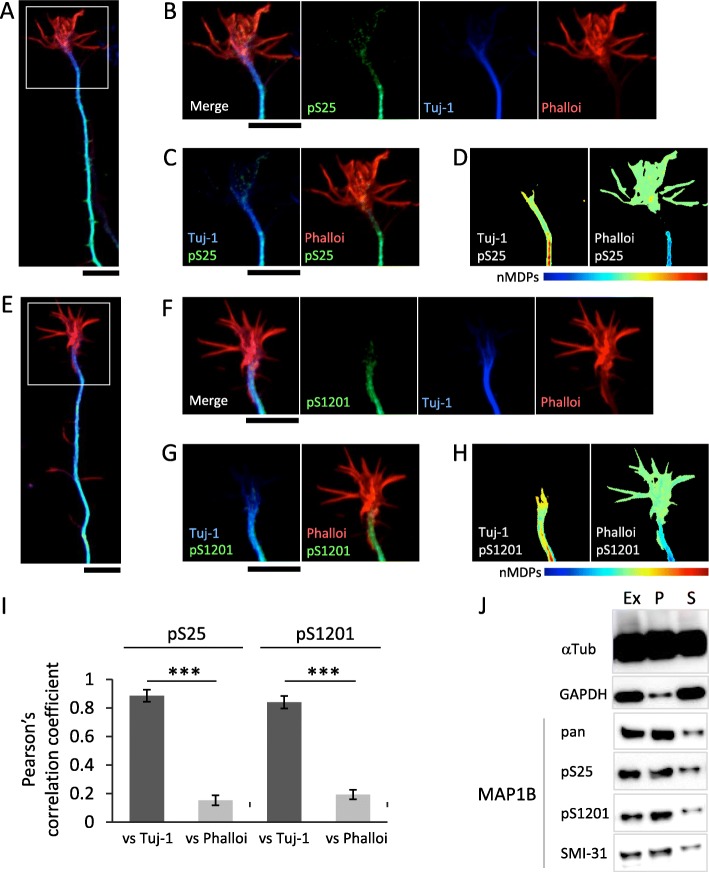
pS25- and pS1201 MAP 1B bind to axonal MTs. **a-h** Immunostaining images of Tuj1 (*blue*) /phalloidin (*red*) /pS25 (*green*: **b-d**), or pS1201 (*green*: **f-h**) in the axonal growth cone of 3DIV mouse cortical neurons. High magnificent images in ROI (**a** or **e**) are shown (**b-d**) or (**f-h**), respectively. Images of normalized mean deviation product (nMDP) (**d**, **(h**; see *Materials and Methods*) from the double staining, Phalloidin/pS25 (**c**) and Tuj1/pS25 (**d**); phalloidin/pS1201 (**g**) and Tuj1/pS1201 (**h**). Tuj1/pS25 and Tuj1/pS1201 show strong co-localization in axons (*cyan* in the merged images and hot colors in the nMDP images), although both phalloidin/pS25 and phalloidin/pS1201 show weak or no colocalization (cold colors in the nMDP images). Scale bars, 3 μm. **i** Quantification of colocalization between the phosphorylated MAP 1B (pS25 or pS1201) and cytoskeletons (tubulin and F-actin) in the axonal growth cone (*n* = 10 for each; ***p < 0.001). Data are presented as mean ± SD. Student’s *t*-test. **j** Determination of MT binding affinities by the co-sedimentation assay, using an extract of E15 mouse brains. Ex: extract; P: pellets; and S: supernatant

Immunoreactivity for both pS25 and pS1201 was distributed similarly to tubulin (Fig. 2a-h), and was quantitatively enriched in the MT area, but not the F-actin area (Fig. 2i). A biochemical co-sedimentation assay for MT binding affinity using an extract of E15 mouse brain showed that immunoreactivity for both Abs was collected in the MT fraction (Fig. 2j), suggesting that these phosphorylation sites are related to the interaction between MAP 1B and MTs. SMI-31 immunoreactivity, which mainly recognizes the phosphorylated neurofilament protein-H and partially recognizes phospho-MAP 1B [22], was similar but not identical to that of pS25 and pS1201, suggesting that SMI-31 has a different specificity from pS25 or pS1201 (Additional file 1: Figure S2).

Using specific inhibitors for c-Jun *N*-terminal kinase (JNK) and glycogen synthase kinase 3β (GSK3β), we determined the responsible protein kinase and found that pS25 was specifically inhibited by JNK inhibitors but not GSK3β inhibitors. In contrast, pS1201 was inhibited by both types of inhibitors (Additional file 1: Fig. S3).

**Fig. 3:**
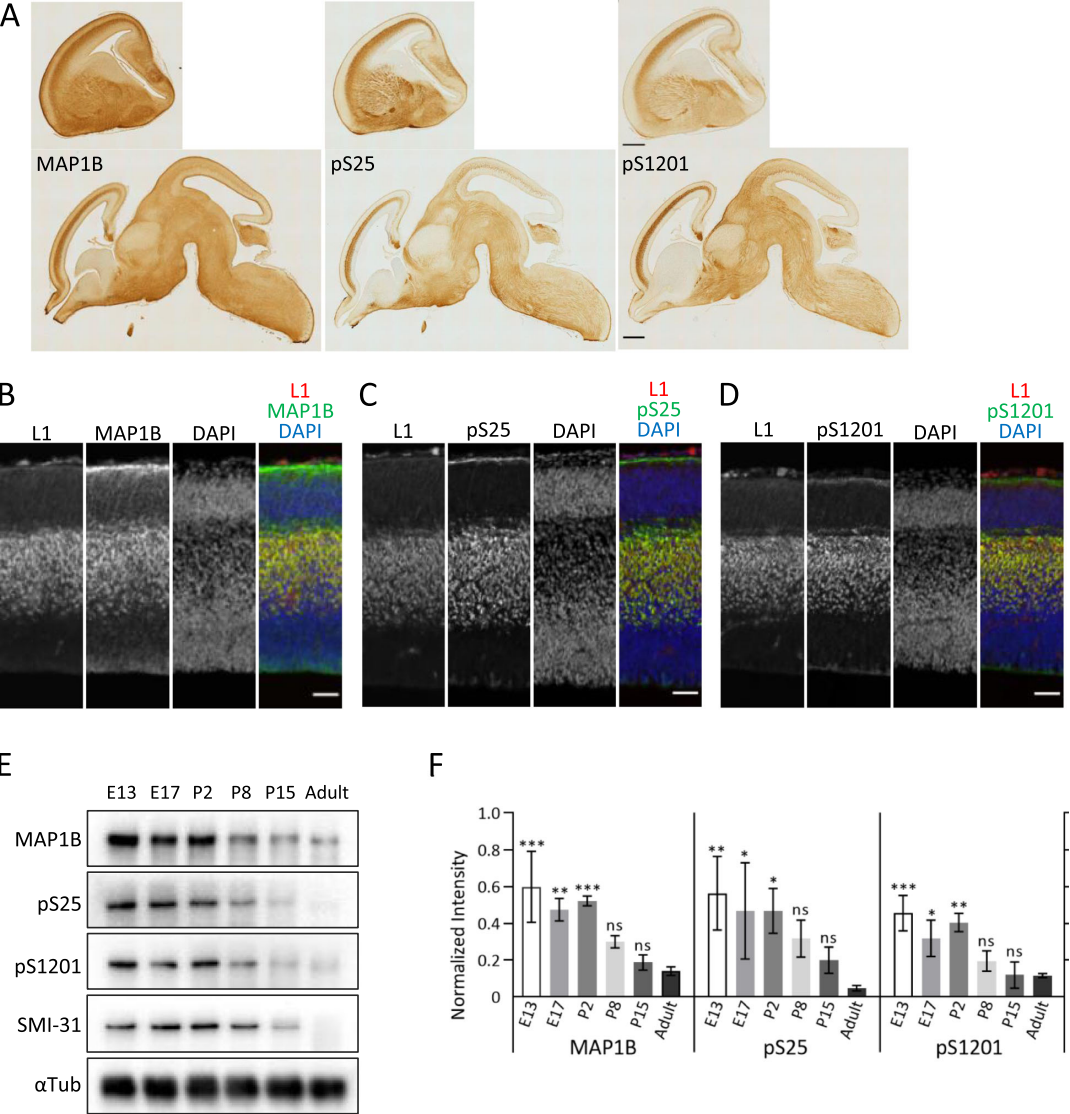
pS25 and pS1201 are spatiotemporally regulated in mouse developing brain. **a** Immunohistochemistry of E15 mouse brain sagittal section using pan-MAP 1B, pS25 and pS1201 Abs, respectively. Scale bar: 500 μm. **b**, **c** and **d** Immunofluorescent staining of E15 neocortex using the Abs against cell adhesion molecule L1 (*red*), as axonal marker [21]; and pS25 or pS1201 (*green*). The neuronal cell nuclei were recognized using DAPI (*blue*). Although expression of MAP 1B were detected in cortical plate and ventricular zone, the staining of pS25 and pS1201 Abs was mainly restricted to the intermediate zone, similar to L1. Scale bar: 50 μm. **e** The expression patterns of MAP 1B, pS25, pS1201 and SMI-31 at developmental stages and in adult mouse brain, evaluated by western blotting. **f** Quantitative assessment of blot intensity showed higher phosphorylation level at a perinatal stage than in adulthood. Data are shown as mean ± SD; *n* = 3; * *p* < 0.05, ** *p* < 0.01, *** *p* < 0.001 by one-way *ANOVA* with Dunnett’s multiple comparisons test

These Abs also preferentially recognized bundles of axons with in vivo immunohistochemistry of sagittal sections of E15 mouse brain (Fig. 3a). Compared to the L1 Ab and DAPI reactivity, pS25 and pS1201 Abs labeled developing axons (Fig. 3b-d). Immunoreactivity of each Ab against pS25 and pS1201 was mainly and highly expressed at developmental stages and rapidly decreased in the mature brain (Fig. 3e-f).

**Fig. 4:**
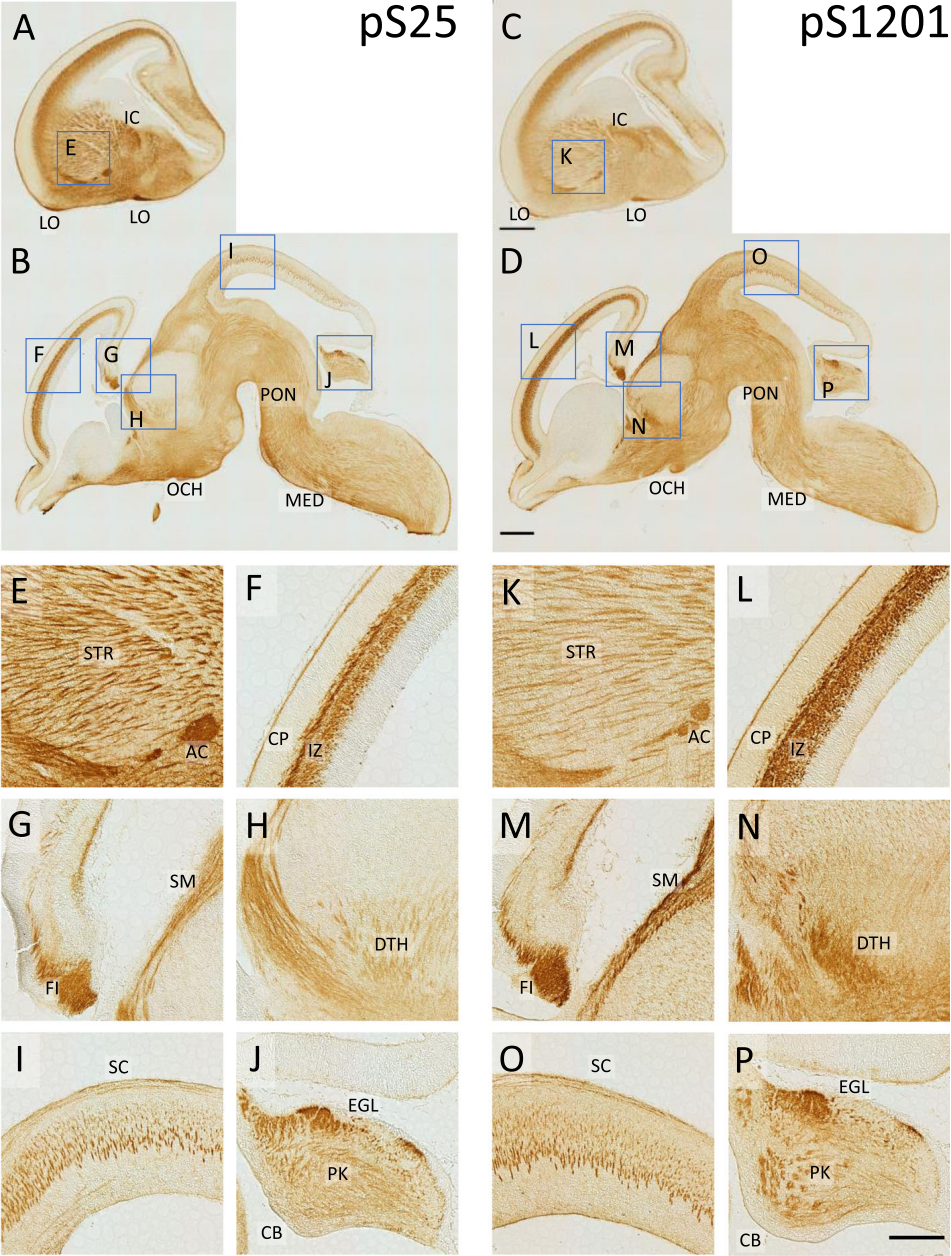
Immunohistochemistry of various brain regions in E15 mouse, using pS25 and pS1201 Abs. Microscopic images of sagittal sections derived from various regions were DAB-stained using pS25 (**a**, **b**, **e-j**) or pS1201 Abs (**c**, **d**, **k-p**). Boxes in (**a-d**) represent the regions enlarged in (**e-p**), respectively. Both pS25 and pS1201 Abs succeeded in labelling the bundles of nerve fibers, such as internal capsule, lateral olfactory tract (**a**, **c**), optic chiasm (**b**, **d**), anterior commissure (**e**, **k**), fimbria of hippocampus, and stria medullaris (**g**, **m**). In the neocortex, the intermediate zone, where the developing axons are enriched, was specifically labelled (**F**, **L**). Fibers in striatum (**e**, **k**), dorsal thalamus (**h**, **n**), superior colliculus (**i**, **o**), and cerebellum (**j**, **p**) were also labelled. The scale bar: 500 μm (**c**, **d**; in **a-d**), 200 μm (**p**; in **e-p**), respectively. *Abbreviations*: *AC*, anterior commissure; *CB*, cerebellum; *CP***,** cortical plate; *DTH*, dorsal thalamus; *EGL*, external granular layer; *FI*, fimbria of hippocampus; *IC*, internal capsule; *IZ*, intermediate zone; *LO*, lateral olfactory tract; *MED*, medulla; OCH, optic chiasm; *PK*, Purkinje cell layer; *PON*, pons; *SC*, superior colliculus; *SM*, stria medullaris; *STR*, striatum

In various regions of the embryonic mouse brain, these Abs also labeled growing axon bundles in vivo (Fig. 4). Taken together, these MAP 1B phospho-specific Abs specifically label growing axons at various stages of development.

### pS25 and pS1201 are maintained in regenerating axons after sciatic nerve injury

**Fig. 5:**
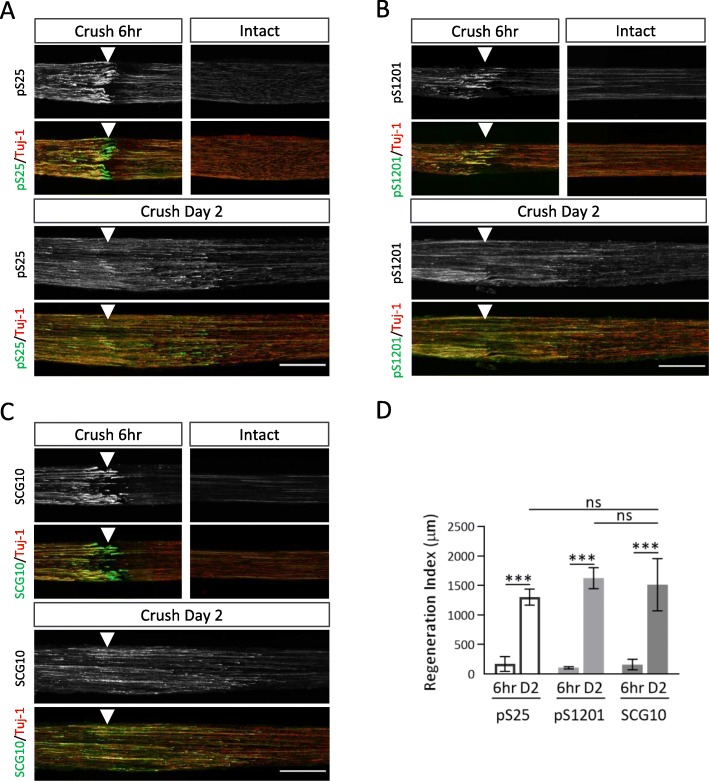
Phosphorylation of S25 and S1201 are involved in the peripheral nerve injury in adult mouse. **a**, **b and c** Immunofluorescence of mouse longitudinal sciatic nerve sections, using pS25 (**a**), pS1201 (**b**), or SCG10 (**c**) Abs (each *green*) and Tuj-1 (*red*) Ab, at 6 h or in 2 days after injury. The proximal side (*left*) and the injured point (*arrowhead*) are shown. *Intact*: sham operation in the other side of the injured nerve. Scale bar: 500 μm. **d** Regeneration index [23], which is the distance from crush site to the location that the signal level of target object decreases to the half of the crush site, of pS25 and pS1201 was higher on 2 days after crush than on 6 h after crush. The index of pS25 and pS1201 was similar to the index of SCG10. *N* = 3 (6 h) and *n* = 3 (2 d); ***p < 0.001 by one-way *ANOVA* with Sidak’s multiple comparisons test

Next, we examined whether these phosphorylation sites serve as molecular markers of regenerating axons as well as GAP-43 pS96 [21]. We chose the crushed sciatic nerve injury as a model of axon regeneration [21]. As a positive control, we used immunoreactivity for SCG10, a protein that inhibits tubulin polymerization in developing neurons. Six hours after the crush injury, both phosphorylated S25 and S1201 were upregulated in the injury site and were co-expressed with SCG10 (Fig. 5a-c). Two days after injury, both areas of phosphorylation had extended to the distal side past the injury point, similar to SCG10 Ab immunoreactivity (Fig. 5a-c). Quantitative analysis revealed that the area recognized by each phospho-specific Ab was increased by more than 10-fold (Fig. 5d). Although expression of MAP 1B itself was upregulated in the crushed nerves, the variance of the signal intensity was not sufficient for calculating the regeneration index (Additional file 1: Figure S4).

**Fig. 6:**
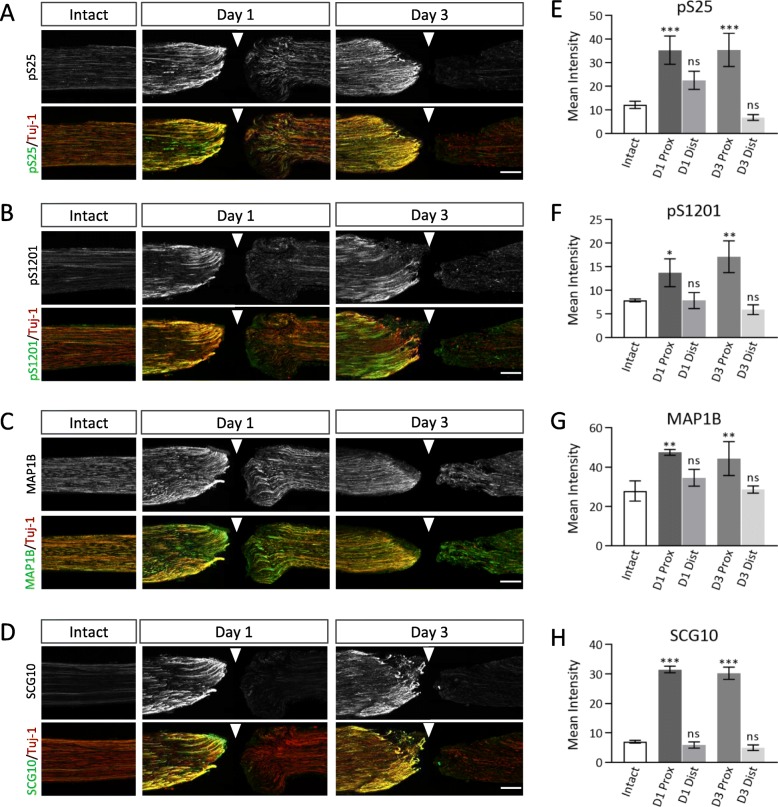
MAP 1B phosphorylation at S25 and at S1201 are induced and maintained in the proximal segment of the transected sciatic nerve. **a**, **b**, **c** and **d** Immunofluorescent staining for pS25 (**a**), pS1201 (**b**), pan-MAP 1B (**c**), or SCG10 (**d**) with Tuj-1 Abs of intact nerve (*left*), transected nerve on 1 day (*middle*), and 3 days (*right*) after injury. On day 1 and day 3, the proximal and the distal segments were located in the left and the right side from the injury point. Scale bar; 200 μm. **e**, **f**, **g** and **h** Quantitative assessment of the signal intensity of pS25 (**e**), pS1201 (**f**), pan-MAP 1B (**g**), and SCG10 (**h**). *N* = 3; * *p* < 0.05, ** *p* < 0.01, and *** *p* < 0.001, by comparison with that in the intact nerve; using one-way *ANOVA* with Dunnett’s multiple comparisons test

We analyzed the distribution patterns of these phosphorylated sites using a different method of injury, namely, transection of the sciatic nerve (Fig. 6).

On day 1 after injury, the proximal segments of the transected nerves showed high immunoreactivity with pS25 and pS1201 Abs compared to the distal segments and the intact nerves (Fig. 6a-b). On day 3, high immunoreactivity for pS25 and pS1201 was maintained in the proximal segments (Fig. 6a-b), but not in the distal ones (Fig. 6a-b). In contrast to pS25 and pS1201, immunoreactivity with a pan-MAP 1B Ab showed only a slight change after nerve transection (Fig. 6c). Increased expression of SCG10 was detected in the proximal segment of the transected nerve on day 1 and was maintained on day 3 (Fig. 6d). Similarly, pS25 and pS1201 immunoreactivity, as well as MAP 1B itself, was maintained (Fig. 6a-c and Fig. 6g), suggesting that the phosphorylation sites at S25 and S1201 in the proximal segment are involved in axon regeneration events. Quantitative analysis confirmed this hypothesis (Fig. 6e, f, and h).

**Fig. 7:**
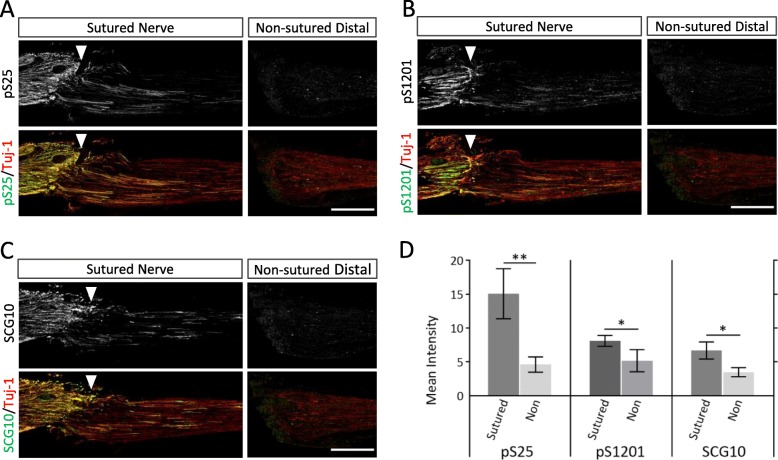
MAP 1B phosphorylation at S25 and S1201 are strongly associated in the regenerating axons. **a**, **b**, and **c** Immunofluorescent staining for pS25 (**a**), pS1201 (**b**), or SCG10 Abs (**c**), comparing with Tuj-1 Ab of the transected and sutured nerves (*left*) and the distal segment of non-sutured, transected nerve (*right*) on 5 days after injury. Scale bar; 500 μm. *Arrowheads* indicate the sutured sites. **d** Quantitative analysis of the signal intensity for pS25, pS1201 and SCG10 Abs, compared to that in the distal segments of the sutured nerve with the transected and non-sutured one. *N* = 3; * *p* < 0.05; ** *p* < 0.01; by unpaired *t* test

Finally, we examined the effects of sciatic nerve injury caused by suturing (Fig. 7). The sutured nerves showed stronger immunoreactivity with pS25 and pS1201 Abs than the non-sutured distal segment (Fig. 7a-b), similar to the SCG10 Ab (Fig. 7c). Namely, in the distal segments of the ligated nerves, the regenerating axons that had penetrated through the repair site were labeled with pS25 and pS1201 Abs (Fig 7a-b). The immunoreactivity with each phospho-specific Ab was significantly more concentrated in the sutured distal segment than in the non-sutured segment (Fig. 7d). These results suggest that the phospho-specific Abs against pS25 and pS1201 of MAP 1B specifically label regenerating axons.

## Discussion

The MAP 1B phosphorylation sites S25 and S1201 are newly characterized in this paper. These two sites were identified by our phosphoproteomics analysis as highly frequent sites among ~ 5000 identified ones [21]. However, the significance of phosphorylation at these sites had not been examined well. Developmentally, such mitogen-activated protein kinase signaling pathways are involved in axon formation via MAP 1B phosphorylation [24–26], suggesting that these new phosphorylated sites were worth examining for a relationship to axon development. In addition, because of our GAP-43 S96 studies [21], we suspected that these sites are likely to be involved in axon regeneration in the adult.

### pS25 and pS1201 of MAP 1B during development

Compared with MAP 1B itself, these phosphorylation sites were enriched in distal axons and growth cones (Fig. 1a-b), suggesting that S25 and S1201 phosphorylation sites are involved in the localization of MAP 1B at the distal portions of the axon in developing neurons. These two sites were distributed similarly to each other according to quantitative analysis (Fig. 1d-g).

The phosphorylation sites, S25 and S1201, are located within the actin-binding domain and the MT-assembly helping domain, respectively [12]. The former domain is thought to be related to the association between MTs and F-actin, and the latter is closely involved in stabilization of MTs [12]. Thus, we examined whether these sites are related to MTs or F-actin bundles. Quantitative analysis revealed that these phosphorylation sites were colocalized with MTs (Fig. 2c and g), rather than F-actin (Fig. 2d and h; also see Fig. 2i), and they co-sedimented with MTs (Fig. 2j), indicating that pS25 and pS1201 are involved in MT binding, but not interactions with F-actin.

Phosphorylation of S25 and S1201 were developmentally regulated and mainly expressed at embryonic stages (Fig. 3e-f), although MAP 1B itself is also expressed during these stages. When considering the distribution and developmental expression patterns, S25 and S1201 phosphorylation seems consistent with the previously described “Mode I”, which was reported to be reduced during development more than two decades ago [18, 27, 28]. “Mode I” phosphoproteins are also localized in the distal portion of axons and growth cones, features that are totally consistent with S25 and S1201 (Fig. 3a-b). SMI-31, which previously was used to characterize “Mode I” phosphorylation, is a monoclonal Ab that mainly recognizes phosphorylation of the high-molecular-weight subunit of neurofilament protein in the adult [22, 29], but also recognizes phosphorylated MAP 1B [30, 31]. The distribution of SMI-31 immunoreactivity was similar but not identical to that with pS25/pS1201 Abs (Additional file 1: Figure S2C-D), and the developmental patterns of SMI-31 were not equal to those of pS25/pS1201 Abs (Fig. 2e), suggesting that SMI-31 recognizes a different epitope than these phosphorylated MAP 1B Abs. Since the previous studies using SMI-31 for Mode-I phosphorylation mainly used the rat brain [27, 28] and ours uses the mouse, there seem the spatial and temporal discrepancies between them due to the species difference. In addition, further investigations will be needed to examine whether SMI-31 also recognize these phosphorylation sites.

We previously demonstrated that JNK is responsible for phosphorylation of P-directed substrates in growth cones, which occupy a large proportion of phosphorylation sites in growth cones as revealed by phosphoproteomics [21]. Here, we used several inhibitors of both JNK and GSK3β.As a result, phosphorylation of S25 was specifically inhibited by JNK inhibitors but not GSK3β inhibitors. However, phosphorylation of S1201 was inhibited by both types of inhibitors (Additional file 1: Figure S3), suggesting that JNK phosphorylates both phospho-sites but GSK3β is involved in only S1201 phosphorylation at developmental stages. This is consistent with previous reports on growing/regenerating axons [32, 33].

Growing axons were specifically labeled by pS25 and pS1201 Abs with in vivo immunohistochemistry in various regions of the developing brain (Fig. 3a-d and Fig. 4). Judging from the phosphoproteomics data of GCM showing that S25 and S1201 are highly frequent sites of MAP 1B phosphorylation [21], these results indicate that S25 and S1201 are the major sites of “Mode I” phosphorylation in vivo*,* and that as for the phospho-specific Abs, which recognize the specific phosphorylation sites as their specified epitopes (Additional file 1: Figure S1B), pS25/pS1201 Abs are much better markers for “Mode I” than SMI-31.

### pS25 and pS1201 of MAP 1B during axon regeneration

Axon regeneration is an important event during repair after injury, and whether mammalian axons can be successfully regenerated or not is an important medical problem [34]. We have postulated that GAP-43 pS96 Ab is a molecular marker of both growing and regenerating axons [21, 35] using the sciatic nerve injury model. Including our previous results that these phosphorylation sites were enriched in GCM, because MAP 1B phosphorylation is related to axon regeneration [36, 37], we suspected that pS25/pS1201 Abs would be potential markers of regenerating axons.

Crushed axons in the sciatic nerve were labeled by these two phospho-specific MAP 1B Abs past the injury point (Fig. 5), as well as by the SCG10 Ab, suggesting that these phosphorylation sites are in part related to axon regeneration. In addition, phosphorylated forms of these sites were concentrated in the proximal end of the transected sciatic nerve (Fig. 6a-b, e-f), as was SCG10 (Fig. 6d and h), implying that the injury response may be transferred to the cell body and that phosphorylated MAP 1B may undergo anterograde axonal transport toward the injury site (Fig. 6a-b).

The sutured nerve experiments revealed that these phosphorylated forms of MAP 1B moved distally past the injury point (Fig. 7a-b, d). These forms may be transported anterogradely and be closely involved in axon regeneration, as is SCG10, a molecular marker of this event (Fig. 7c-d). These phosphorylated forms in the injury experiments may selectively undergo anterograde axonal transport to stimulate MT synthesis and stabilization in regenerating axons [38].

SCG10, a member of stathmin family, forms T_2_S (tubulin dimer-stathmin) complexes and sequesters tubulin, and subsequently, SCG10 inhibits MT formation [39, 40]. SCG10 is related to axon growth via its ability to destabilize MTs [41–43] and is enriched in growth cones, as shown using proteomics [1–3]. Both SCG10 and pS25/pS1201 of MAP 1B were involved in axon regeneration (Figs. 5-7), suggesting that MT remodeling by these proteins in both directions may facilitate this event.

A regeneration index was measured based on SCG10 immunoreactivity to evaluate axon regeneration [23]. The regeneration indices of pS25 and pS1201 were similar to that of SCG10 (Fig. 5). Thus, these two phospho-specific Abs against MAP 1B may be useful for measuring the “regeneration index” [23]. SCG10 is expressed and highly phosphorylated mainly in developmental stages [21, 44], depolymerizes MTs, and regulates them in an opposite manner than MAP 1B. However, both are necessary for axon development and probably regeneration. Dynamic MTs are thought to be essential to growing/regenerating axons, and these two proteins are needed for dynamic regulation of MTs in both directions [45, 46]. Judging from our results, pS25 and pS1201 are likely to be involved in MT dynamics, and in the next step, how these two phosphoproteins, MAP 1B and SCG10 dynamically regulate MT organization in the growing axon, should be directly elucidated.

Finally, the functional understanding of these phosphorylation sites still remained to be elucidated. We showed here that phosphorylated forms at S25 and S1201 of MAP 1B are enriched in the growth cone and the distal axons (Figs. 1-3), and regulated during development (Fig. 4), suggesting that phosphorylation at these sites will greatly contribute to rapid axon formation, including after injury when axon regeneration is required.

As a next step, the molecular interactions that occur via these sites and colocalization of these molecules with phospho-MAP 1B should be clarified to increase our understanding of the regulatory mechanisms of axonal MT dynamics in these events. Superresolution microscopic techniques and the use of our Abs will increase our understanding of how MTs (and F-actin) are regulated in growth cones when axons grow due to phosphorylation at these sites of MAP 1B [1, 4, 47, 48]. In addition, evolutionary analysis of the phosphorylation sites should elucidate important aspects of each phosphoprotein and phosphorylation site and will be helpful for further functional analysis of them [49, 50].

## Materials and methods

### Animals

All of the animal experiments were performed following approval from the Animal Resource Center of Niigata University. Pregnant ICR mice were purchased from Japan-SLC, Inc. (Shizuoka, Japan) and used for neuronal cell culture, immunostaining of embryonic mouse brain, and analysis of protein expression and phosphorylation levels in developing brain. Adult C57BL/6 N mice were used for the experiments with sciatic nerve injury.

### Abs

The Abs used in this paper and their dilutions are listed in Additional file 2: Table S1. The pS25 and pS1201 Abs were produced as previously described [21].

### Plasmid construction

Inverse PCR-based mutagenesis techniques for the addition of a 2 × FLAG (DYKDDDDK) tag to the *N*-terminus of the rat *MAP 1B* sequence in the pMT5CMV vector [51, 52], a gift from Dr. F. Propst (Department of Biochemistry and Cell Biology, Max F. Perutz Laboratories, University of Vienna, Vienna, Austria), were performed using the KOD–Plus-Mutagenesis Kit (Toyobo Co., Ltd., Osaka, Japan) and the following primers: 2xFlag-pMT5-F (5′-ATGATGGATTACAAGGATGACGACGATAAGGATTACAAGGACGACGACGACAAGGGACTAGTCGCCACC-3′) and 2xFlag-pMT5-R (5′-AGTCAATTGTCGACGCGGCCGC-3′). To generate phosphorylation site mutations of *MAP 1B* (S25A, S1201A), inverse PCR-based mutagenesis techniques were also performed using the following primers: S25A sense (5′-ACCGCACCCAGCCTGTCGCAC-3′); S25A antisense (5′-GGTCGCCGCCGGGTTGC-3′); S1201A sense (5′-ATAGCACCACCTTCGTCCATGGAAGAAGAC-3′); and S1201A antisense (5′-GGTGGAGGCGGAAGCGTTGTAATC-3′).

### Transfection of COS-7 cells and immunoprecipitation assay

Transfection experiments using COS-7 cells were performed as described previously [5]. Each plasmid expressing 2 × FLAG-tagged wild type, S25A, or S1201A full-length *MAP 1B* were transfected using PEI ‘MAX’ (Polysciences, Inc., Warrington, PA, USA). After 48 h, the transfected cells were lysed with lysis buffer (1% NP-40, 50 mM Tris-HCl [pH 7.5], 150 mM NaCl) containing protease inhibitors (10 μg/ml leupepsin, 10 μg/nl pepstatin A, and 0.1 mM p-APMSF) and phosphatase inhibitors (20 mM NaF and 1 mM Na_3_VO_4_), sonicated, and centrifuged at 12,000×*g* at 4 °C. To each supernatant, 2 μg anti-DDDDK-tag mAb (clone FLA-1, Medical & Biological Laboratories Co., Ltd., Aichi, Japan) was added, and the mixture was incubated at 4 °C for 3 h. In addition, 25 μl Protein G Mag Sepharose slurry (GE Healthcare UK, Ltd., Buckinghamshire, UK) was mixed and incubated at 4 °C for 1 h and eluted with 1 × sample buffer for SDS-PAGE.

### Western blotting and analysis of the MAP 1B phosphorylation levels

Protein samples were separated by SDS-PAGE in 6% gel or 4–20% polyacrylamide gradient gel, soaked in transfer buffer and electroblotted onto PVDF membrane overnight. The membrane was incubated with primary Abs for 90 min and with HRP conjugated secondary Abs for 30 min at room temperature. Protein bands were visualized with an ECL Prime kit (GE Healthcare UK, Ltd.). For developmental analysis of MAP 1B, the whole brains of E13, E17, postnatal day (P) 2, P8, P15 and adult mice were homogenized in lysis buffer with protease inhibitors and phosphatase inhibitors and centrifuged at 12,000×*g* at 4 °C. Half volume of 3 × sample buffer for SDS-PAGE was added to each supernatant. To analyze the expression or the phosphorylation level of MAP 1B, ImageJ software Fiji (http://rsb.info.nih.gov/ij) was used to measure the areas under the curve of the immunoblotted bands. In the developmental analysis, the values were normalized to α-tubulin Ab and compared to that of adult brain, which was defined as 1.0.

### Neuronal cell culture

The cerebral cortex neurons of E15 mice were cultured, as described previously [4, 6, 21]. For the inhibition assay of MAP 1B phosphorylation, 4 days in vitro (DIV) cortical neurons were treated for 24 h with culture medium containing DMSO as a control, one of three JNK1 inhibitors [SP600125, JNK Inhibitor V, and JNK Inhibitor XVI (Cayman Chemical, USA)], or one of three GSK3β inhibitors [IM-12, TDZD-8 (Cayman Chemical, USA), and LiCl (99.5% purity, Wako, Osaka, Japan)]. Cells exposed to each inhibitor were lysed in 1 × SDS-PAGE sample buffer and processed for western blotting analysis.

### Immunofluorescent staining and image analysis

The 3–4 DIV cortical neurons were fixed for 15 min with 4% paraformaldehyde (PFA) in PBS, permeabilized using 0.1% TritonX-100 in PBS and incubated in 3% BSA/PBS for blocking. The cells were incubated with primary Abs diluted in 1% BSA/PBS overnight at 4 °C, and then incubated with secondary Abs (Jackson ImmunoResearch Inc., West Grove, PA, USA; 1/1000 dilution), for 30 min at room temperature, and mounted in Fluorescence Mounting Medium (Agilent Technologies, Inc., Santa Clara, CA, USA). Samples were observed using a confocal microscope (FV1200; Olympus, Tokyo, Japan). For the line plot analysis of axons, the longest neurite defined as an axon, and the second longest neurite, defined as dendrite were outlined with the ROI tool of ImageJ. The mean intensities of the fluorescence in each ROI were measured. The immunopositive ratios of pS25 or pS1205 Abs to pan-MAP 1B Ab in an axon and a dendrite were calculated, and the ratios of axon was compared to that of dendrite, which was defined as 1.0. For the colocalization assay, values of either the normalized mean deviation product or Pearson’s correlation coefficient (R value) were calculated with the plug-ins of ImageJ/Fiji software, Colocalization Colormap [53] or coloc2, respectively.

### MT co-sedimentation assay

The MT co-sedimentation assay was performed according to the method described in [54] with modifications. E15.5 mouse brains were homogenized in assembly buffer (100 mM MES, 0.5 mM MgCl_2_, 1 mM EGTA, pH 6.8) containing 1 mM DTT, 1 mM APMSF, 1 μg/ml pepstatin A, 1 μg/ml leupeptin, 1 mM Na_3_VO_4_, 1 mM NaF, 1 mM Na_2_MoO_4_, and 2 mM imidazole, with a glass-Teflon homogenizer. The homogenate was centrifuged at 30,000×*g* for 1 h at 4 °C, and crude extract was mixed with glycerol (1/3 volume of the total extract) and 1 mM GTP (final concentration). After incubation at 37 °C for 40 min, MTs and MT-associated proteins were collected by centrifugation at 100,000×*g* for 40 min at 37 °C. The pellets were then re-suspended in the assembly buffer with the same volume as the supernatant.

### Immunohistochemistry of mouse embryonic brain

Immunohistochemistry with phospho-specific MAP 1B Abs on embryonic mouse brain was performed as described previously [21]. In brief, brains of E15 mice were fixed with 4% paraformaldehyde in 0.1 M phosphate buffer (pH 7.4) for 1 d at 4 °C and cryoprotected with 30% sucrose in 0.1 M phosphate buffer. Specimens were immersed in a solution consisting of OCT compound (Sakura Finetechnical Co., Ltd., Tokyo, Japan) and 30% sucrose/0.1 M phosphate buffer and embedded by freezing in ethanol cooled with dry ice. Sagittal sections were sliced at a thickness of 20 μm using a sliding cryotome (CM1850; Leica Biosystems, Wetzlar, Germany) and thaw-mounted on MAS-coated slide glass (Matsunami Glass Inc., Ltd., Osaka, Japan).

For diaminobenzidine staining, slides were incubated with methanol containing 0.3% H_2_O_2_ for 30 min, washed with 0.2% Triton X-100 in PBS (PBST), and then incubated overnight with primary Abs diluted in 1% BSA/PBST. On the next day, the slides were reacted with *N*-Histofine Simple Stain Mouse MAX PO (R) (Nichirei Biosciences Inc., Tokyo, Japan), and brown color was developed using a diaminobenzidine substrate kit (Nichirei Biosciences Inc.). Images were acquired with an upright microscope (BX63; Olympus) equipped with differential interference contrast optics.

For multiple fluorescent staining, slides were incubated overnight with primary Abs diluted in 1% BSA/PBST, and then incubated with Alexa Fluor 488 Goat Anti-Rabbit IgG (H + L) Ab (1/500), Goat anti-Rat IgG (H + L) Cross-Adsorbed Secondary Ab, Alexa Fluor 594 (Life Technologies; 1/500), and 4′, 6-diamidino-2-phenylindole, dihydrochloride (DAPI; Life Technologies; 1/5000). Fluorescent images were acquired with an upright microscope (BX63; Olympus).

### Sciatic nerve injury

Female C57BL/6 N mice (3–5 months old) were anesthetized by intraperitoneal injection of a mixture of ketamine, xylazine, and pentobarbital, and the following three types of injury models were made: (Model 1) Using the protocol reported previously [23, 55], the right sciatic nerve was crushed with a fine forceps (Fontax, INOX #5) for 30 s, and the mice were sacrificed 6 h or 2 d after the operation. (Model 2) The right sciatic nerve was transected with surgical scissors, and the mice were sacrificed 1 or 3 d after the operation. (Model 3) The right sciatic nerve was transected and then repaired by end-to-end suturing using 10–0 nylon, and the mice were sacrificed after 5 d. In all three models, the left sciatic nerve underwent a sham operation. Three mice were used for each surgery.

For immunohistochemistry, the sciatic nerves were treated as described previously [21]. Alexa Fluor 488 Goat Anti-Rabbit IgG (H + L) Antibody (Jackson ImmunoResearch Inc.) and Streptavidin Alexa Fluor 594 conjugate (Life Technologies; 2 μg/mL) were used as the secondary Abs. To evaluate the regeneration index [21, 23], the intensity along the nerve was measured using a rectangular ROI with ImageJ. To measure the signal intensity, the nerve was outlined with the freehand ROI tool of ImageJ so that the area was 0.4 mm^2^, and the mean intensity was measured. The regeneration index [23], which is the distance from the crush site to the location that the signal level of the target object decreases to half of that of the crush site, was calculated.

### Statistics

All data are represented as mean values ± standard deviation (SD). Paired or unpaired Student’s *t* tests or one-way analysis of variance (*ANOVA*) with Dunnett’s or Sidak’s post-hoc multiple comparison tests were performed using GraphPad Prism7 (GraphPad Software), and *p* < 0.05 was considered to be statistically significant.

## Supplementary information

**Additional file 1: Figure S1.** The specificity of pS25 and pS1201 Abs. **Figure S2.** pS25 and pS1201 Abs labeled more specific parts of the axon than SMI-31 Ab. **Figure S3.** Inhibitor sensitivities of pS25 and pS1201. **Figure S4.** Fluorescent immunostaining of pan-MAP 1B in the sciatic nerve.

**Additional file 2: Table S1.** Abs used for immunodetection in this paper.

## Data Availability

The phosphopeptides identified using phosphoproteomics for MAP 1B are shown in ref. [21] and its supplementary information files.

## Abbreviations

Ab: Antibody
ANOVA: Analysis of variance
BSA: Bovine serum albumin
GCM: Growth cone membrane
GSK3β: Glycogen synthase kinase-3β
JNK: C-jun *N*-terminus kinase
MAP 1B: Microtubule-associated protein 1B
PBS: Phosphate-buffered saline
ROI: Region of interest
SD: Standard deviation
