# Supplementary figures and images for: Phosphorylation sites of microtubule-associated protein 1B (MAP 1B) are involved in axon growth and regeneration
Source: Mol Brain. 2019 Nov 11;12:93. doi: 10.1186/s13041-019-0510-z (PMC6849251; doi:10.1186/s13041-019-0510-z)

Figure S1

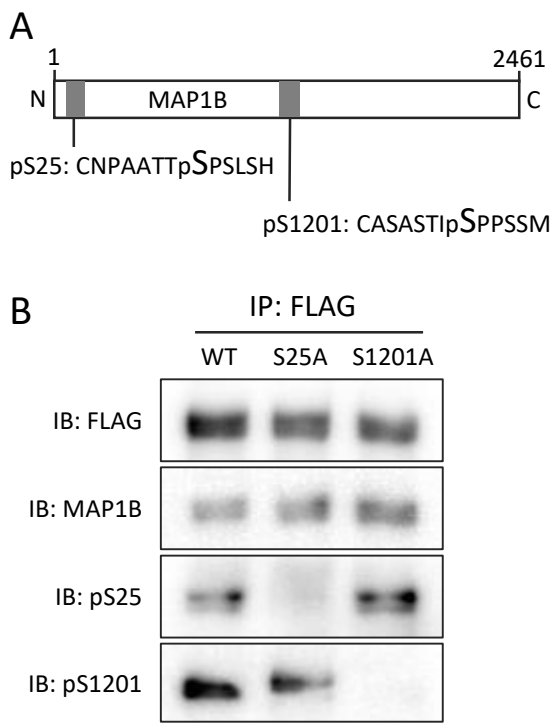

Figure S2

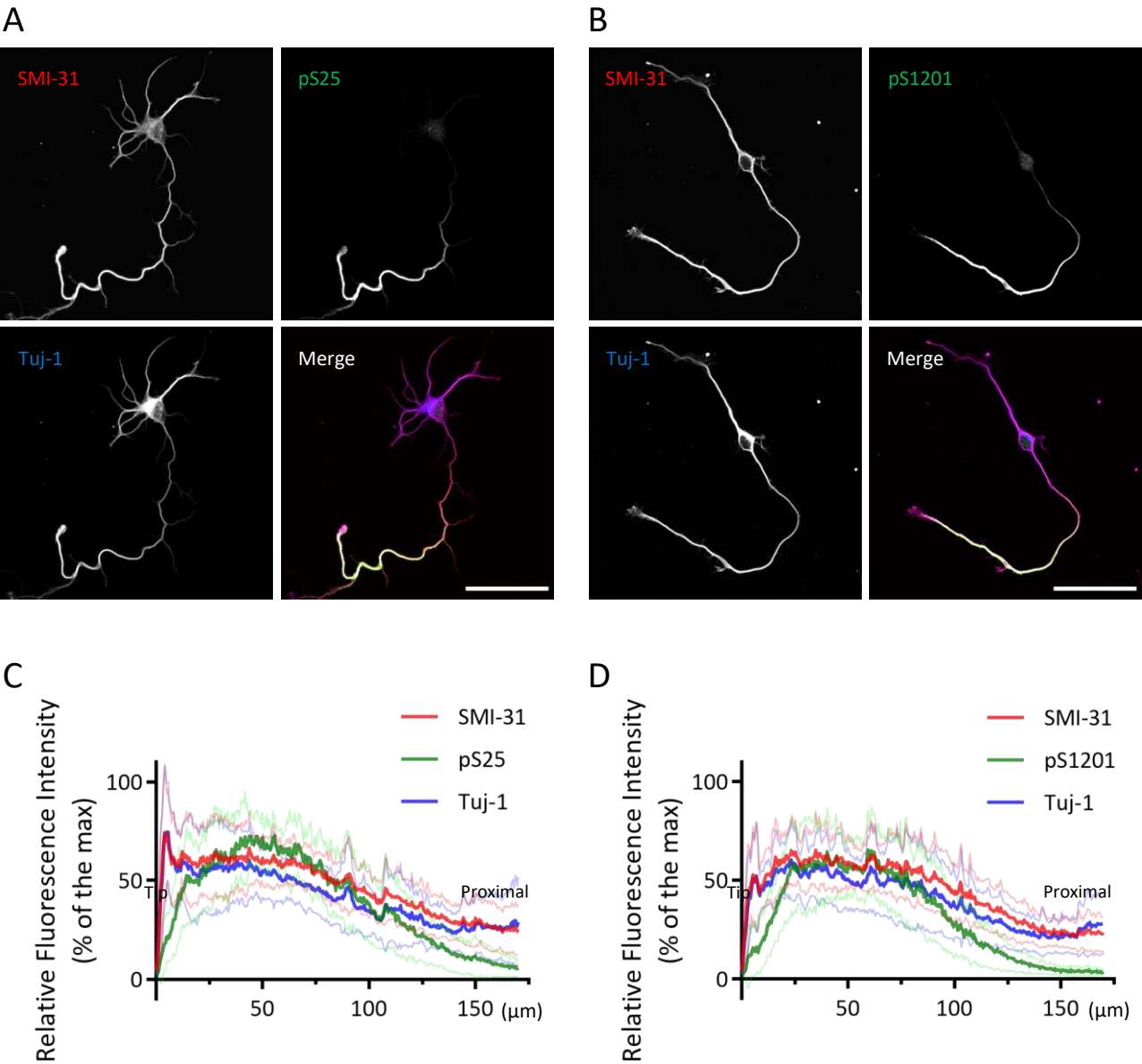

Figure S3

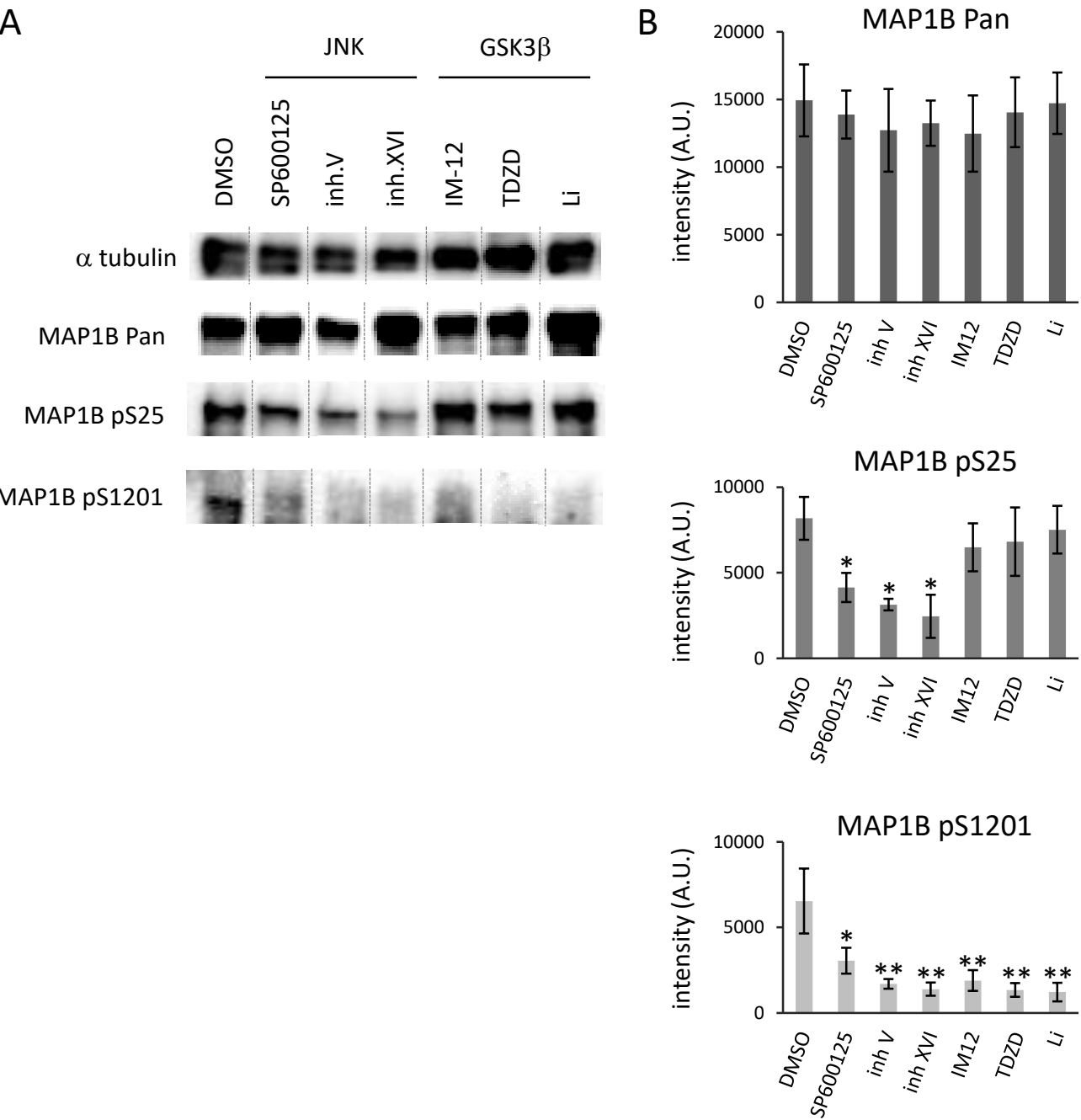

Figure S4

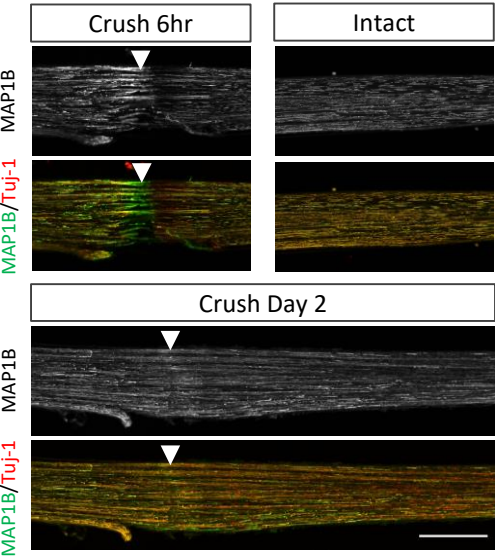

Supplement: Supplementary file 1 — Additional file 1: Figure S1. The specificity of pS25 and pS1201 Abs. Figure S2. pS25 and pS1201 Abs labeled more specific parts of the axon than SMI-31 Ab. Figure S3. Inhibitor sensitivities of pS25 and pS1201. Figure S4. Fluorescent immunostaining of pan-MAP 1B in the sciatic nerve. [file 13041_2019_510_MOESM1_ESM.pdf]
