# Supplementary material for: Phosphorylation sites of microtubule-associated protein 1B (MAP 1B) are involved in axon growth and regeneration
Source: Mol Brain. 2019 Nov 11;12:93. doi: 10.1186/s13041-019-0510-z (PMC6849251; doi:10.1186/s13041-019-0510-z)
Supplement: Supplementary file 2 — Additional file 2: Table S1. Abs used for immunodetection in this paper. [file 13041_2019_510_MOESM2_ESM.docx]

| **Ab** | **Supplier** | **Species** | **Dilution** | | |
| --- | --- | --- | --- | --- | --- |
|  |  |  | **WB** | **IF** | **IHC** |
| MAP1B (clone H-8) | Santa Cruz Biotechnology | mouse | − | (C)1:1000 | − |
| MAP1B (#21633-1-AP) | Proteintech | rabbit | 1:1000 | (T)1:200 | 1:50 |
| MAP1B (pS25) | our group | rabbit | 1:2000 | (C)1:2000; (T)1:3000 | 1:3000 |
| MAP1B (pS1201) | our group | rabbit | 1:500 | (C)1:1000; (T)1:200 | 1:200 |
| DDDDK(FLAG)-tag (clone FLA-1) | MBL | mouse | 1:3000 | − | − |
| α-Tubulin (clone DM1A) | Sigma-Aldrich | mouse | 1:20000 | − | − |
| Neural Cell Adhesion Molecule L1 (clone 324) | Merck | rat | − | 1:500 | 1:500 |
| Stathmin-2 (STMN2 or SCG10) (#NBP1-49461) | Novus Biologicals | rabbit | − | 1:3000 | − |
| β-Ⅲ Tubulin (clone Tuj-1) | Covance | mouse | − | (C)1:1000 |  |
| β-Ⅲ Tubulin Biotinylated (clone Tuj-1) | R&D systems | mouse | − | (C)1:1000; (T)1:500 | − |
| Phosphorylated Neurofilament (clone SMI-31) | Covance | mouse | 1:1000 | 1:1000 | − |
| GAPDH | MBL JAPAN | mouse | 1:2000 | − | − |
| GAP-43 (pan) | Millipore | rabbit | 1:1000 | − | − |
| GAP-43 (pT172) | our group | rabbit | 1:1000 | − | − |

**Table S1. Abs used for immunodetection in this paper.**

WB: Western blotting; IF: Immunofluorescence; IHC: Immunohistochemistry; C: cell staining; T: tissue staining
